# Speech under stress: Affective and cognitive reactivity in schizophrenia and their functional correlates^☆^

**DOI:** 10.1016/j.scog.2026.100423

**Published:** 2026-02-14

**Authors:** Kyle S. Minor, Madisen T. Russell, Evan J. Myers, Audrey T. Satchivi, Maya E. Brown-Hughston, Erica L. Whiting, Deborah Daluga, Rachel C. Marks, Michaela M. Di Palmo, Basma O. Aly

**Affiliations:** a Department of Psychology, Indiana University – Indianapolis, Indiana University Indianapolis School of Science, 402 N Blackford St, Indianapolis, IN, 46202, United States; b Boston Medical Center Wellness and Recovery After Psychosis Program, 850 Harison Avenue, Dowling Building, 8^th^ Floor, Boston, MA, 02118, United States; c Department of Psychiatry, Indiana University School of Medicine, 340 W 10^th^ St., Indianapolis, IN, 46202, United States

**Keywords:** Disorganized speech, Schizophrenia, Negative affect, Neurocognition, Social functioning

## Abstract

Disorganized speech is a core diagnostic criterion of schizophrenia, yet mechanisms driving its variability remain unclear. Building on evidence from schizotypy and first-episode psychosis literature, we examined whether affective and cognitive systems influence speech disorganization in schizophrenia. Thirty-five individuals with schizophrenia (n = 35) and 37 healthy controls completed a validated speech paradigm across three conditions: neutral, affective, and cognitive load. Trained raters assessed disorganized speech using the Communication Disturbances Index (CDI). Reactivity was quantified using standardized residual change scores. The schizophrenia group exhibited significantly greater disorganized speech across all conditions (*d* = 0.58–0.89). Of note, affective reactivity emerged only when using regression-based analyses controlling for neutral condition disorganization, not with repeated-measures ANOVA—revealing important methodological considerations for detecting subtle stress-vulnerability patterns. Despite well-documented cognitive deficits in schizophrenia, cognitive reactivity was not observed. Both affective and cognitive reactivity showed medium inverse correlations with neurocognitive functioning (*r* = −0.36 to −0.41), but lower correlations with social or role functioning, contrasting with findings in earlier illness stages. These results demonstrate that disorganized speech in schizophrenia is contextually sensitive, with reactivity patterns linked to cognitive impairment. Furthermore, it builds upon prior evidence in schizotypy and first-episode psychosis groups, establishing associations across the psychosis-spectrum. Future research should explore how reactivity patterns evolve across illness stages to inform tailored interventions targeting emotion regulation and cognitive remediation based on individual reactivity profiles.

## Highlights

• The schizophrenia group showed greater disorganized speech across all conditions.
• Affective reactivity emerged only with regression analyses, not repeated measures.
• Both affective and cognitive reactivity inversely correlated with neurocognition.

## 1 Introduction

We've all had moments where our train of thought derails, the right words vanish, or the thread of what we're saying disappears. While these moments are typically fleeting for most of us, they can be persistent and impairing for people with schizophrenia who experience disorganized speech. Disorganized speech refers to verbal communication that is difficult to follow due to problems with the logical sequencing, coherence, or connectivity of ideas, and it is the primary behavioral manifestation of positive formal thought disorder. As a core diagnostic criterion of schizophrenia (American Psychiatric Association, 2013), disorganized speech has long drawn clinical and scientific attention (Bleuler, 1950; Kraepelin, 1919). In addition to being linked with significant difficulties in social engagement and school/work performance (Cornblatt et al., 2007; Rocca et al., 2018; see Oeztuerk et al., 2022), recent qualitative research has highlighted the impact of disorganized speech on autonomy and daily life: it affects relationships, employment, and independent living (Bettis et al., 2024; Olarewaju et al., 2023). Despite its longstanding importance, there is still much unknown about the mechanisms that exacerbate disorganized speech in schizophrenia.

The diathesis-stress model posits that underlying vulnerabilities manifest under specific environmental, cognitive, or emotional demands (Nuechterlein and Dawson, 1984; Zubin and Spring, 1977), highlighting the importance of understanding the mechanisms that drive disorganization. Consistent with this view, disorganized speech is increasingly recognized as a dynamic phenomenon that varies with emotional state, cognitive load, and social context (Agostini et al., 2021; Docherty, 2005; Kuperberg, 2010; Merrill et al., 2017; Minor et al., 2016; Olarewaju et al., 2023). To assess this variability, researchers use speech paradigms that measure *reactivity*—changes in disorganization from neutral to experimentally manipulated conditions (Minor et al., 2016; Docherty and Hebert, 1997). Reactivity may represent a stable marker of vulnerability to psychosis (Barrantes-Vidal et al., 2013; Myin-Germeys and van Os, 2007) and has been observed across the psychosis-spectrum using speech paradigms (de Sousa et al., 2016; Minor and Cohen, 2010).

Recent neuroimaging work has begun to elucidate the networks underlying disorganization, implicating disrupted connectivity in language and executive control regions (Kircher et al., 2018; Palaniyappan et al., 2015). These findings suggest that the behavioral manifestations of disorganized speech may reflect dysfunction in distributed brain systems that are sensitive to both affective and cognitive demands. Using speech paradigms, two similar mechanisms—negative affect and cognitive load—have emerged as potential drivers of speech reactivity. Evidence for affective reactivity is robust: studies across schizotypy (Marggraf et al., 2019), first-episode psychosis (Minor et al., 2016), and schizophrenia (Burbridge and Barch, 2002; de Sousa et al., 2016; Docherty and Hebert, 1997; Mohagheghi et al., 2013; Rubino et al., 2011) show that disorganized speech intensifies when negative emotions are induced. Qualitative research similarly suggests that specific negative affective themes—such as adverse experiences, alienation, and interpersonal tension—can trigger disorganization (Bettis et al., 2024; Popovic et al., 2019). In contrast, evidence for cognitive load is mixed: some studies report increased disorganization under cognitive challenge (Granholm et al., 1996; Marggraf et al., 2019); others do not (Le et al., 2017; Minor et al., 2016). Nonetheless, compelling theoretical rationale exists for expecting that increased cognitive demands, particularly those taxing working memory, disproportionately affect individuals on the psychosis-spectrum (Chun et al., 2013; Docherty et al., 2003; Kerns and Berenbaum, 2002; Melinder and Barch, 2003).

Beyond understanding affective and cognitive mechanisms in isolation, it is also important to determine how disorganized speech and reactivity are linked to real-world functional deficits in schizophrenia. Meta-analyses report small to moderate associations between disorganized speech and social functioning (de Sousa et al., 2019; Marggraf et al., 2020), and individual studies suggest that both neutral disorganization and affective reactivity may help explain these impairments (Bowie et al., 2011; Minor et al., 2016). Associations with occupational functioning have also been observed across the psychosis-spectrum, including individuals high in schizotypy (Hernández et al., 2023), those with first-episode psychosis (Roche et al., 2016), and people with schizophrenia (Rocca et al., 2018).

To systematically investigate affective and cognitive load, our laboratory developed a speech paradigm that manipulates both affective and cognitive demands (see also Cohen et al., 2009; Minor et al., 2011). We previously used this paradigm with participants high in schizotypy (Marggraf et al., 2019) and in those with first-episode psychosis (Minor et al., 2016). In these studies, disorganized speech increased under specific conditions in the schizotypy group and across all conditions in the first-episode psychosis group. We also found evidence of cognitive reactivity in schizotypy and affective reactivity in first-episode psychosis. Notably, affective reactivity was associated with poorer social functioning in both samples. The goal of the current study was to extend this work by applying the same paradigm to individuals with schizophrenia.

In this study, we had two aims. First, we examined whether disorganized speech was more prevalent for people with schizophrenia compared to controls across neutral, affective, and cognitive conditions, and if affective or cognitive reactivity was evident in the schizophrenia group. Based on prior research across the psychosis spectrum (de Sousa et al., 2016; Minor et al., 2016), we expected elevated disorganization in schizophrenia with evidence of affective but not cognitive reactivity. Second, we determined whether affective or cognitive reactivity was associated with neurocognitive or functional outcomes in schizophrenia. Based on past studies (Bowie et al., 2011; Couture et al., 2006; Minor et al., 2016), we expected disorganized speech and reactivity to be inversely related to social, role, and neurocognitive functioning. Clarifying these associations is important not only for understanding how disorganized speech relates to real-world impairment but also for advancing mechanistic and clinical models of speech.

## 2 Methods

### 2.1 Participants

Participants for this study were recruited through: 1) the All IN For Health database, an online research registry maintained by Eskenazi Health and Indiana University that connects researchers with community members interested in participating in research studies; 2) previous research studies; and 3) a local community mental health center. The study consisted of 35 outpatients with a diagnosis of schizophrenia or schizoaffective disorder and 37 healthy controls closely matched by age, race, ethnicity, and gender. Medical records were reviewed to confirm diagnosis, followed by the administration of the Mini International Neuropsychiatric Interview (MINI, Sheehan et al., 1998). Additional eligibility criteria included age (18–65), clinical stability (i.e., no medication changes or hospitalizations within the last month), and fluency in English. Participants were excluded if they had a current substance use disorder, neurological condition, or intellectual disability.

### 2.2 Measures

#### 2.2.1 Speech paradigm

To investigate affective and cognitive influences on speech, we used a validated paradigm developed by our laboratory that manipulates both affective and cognitive demands (see also Cohen et al., 2009; Minor and Cohen, 2012; Minor et al., 2016). The paradigm consists of three conditions: neutral, affective, and cognitive load. In each condition, participants spoke into a head-mounted microphone for 2 min while their speech was recorded for later transcription and analysis.

**Fig. 1:**
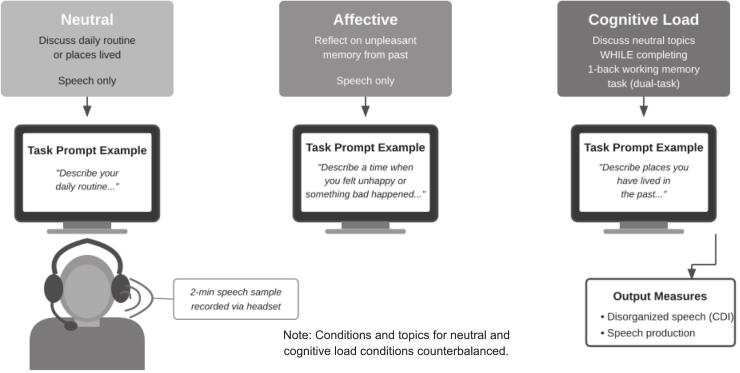
Key characteristics of the speech paradigm.

In the neutral condition, participants discussed either their daily routine or places they had lived. In the affective condition, participants chose an unpleasant memory from their past to reflect on. In the cognitive load condition, participants discussed neutral topics (daily routine or places they lived) while completing a dual-task, one-back visual working memory test. Task prompts were displayed on a computer screen. The order of speech conditions was counterbalanced across participants (see Fig. 1).

The cognitive load condition involved a dual-task one-back visual working memory paradigm. Participants first completed a baseline trial of the visual working memory test alone (without speaking). Then, they completed the dual-task block, in which they spoke continuously for 2 min about the other neutral topic (i.e., daily routine or places they lived, counterbalanced across participants) while simultaneously performing the visual working memory test. During this block, 35 trials were presented on a computer screen. Each trial consisted of a symbol (e.g., “*”, “#”) presented at 1500 ms intervals for up to 2000 ms. Upon symbol presentation, participants were instructed to press the ‘7’ key if the current symbol matched the immediately preceding symbol or the ‘v’ key if it did not.

Two-minute speech samples were selected based on prior work demonstrating that this duration is sufficient to detect disorganized speech across the schizophrenia-spectrum (Minor and Cohen, 2010; Minor et al., 2016; Marggraf et al., 2019). Research assistants transcribed the recorded speech samples verbatim.

#### 2.2.2 Disorganized speech

Disorganized speech was measured using the Communication Disturbances Index (CDI), a well-validated measure with established psychometric properties in schizophrenia populations (Docherty, 1996; Minor and Cohen, 2010). The CDI captures impairments that result from difficulties in tailoring speech to communication contexts and listener needs (Docherty, 2005; Meister et al., 2025). For example, instances of disorganized speech are counted when the true meaning or intended meaning is unclear. The CDI captures multiple dimensions of disorganization, including ambiguous word meanings, confused references, vague references, missing information references, structural unclarities, and wrong word references. Disorganized speech scores were calculated for each transcript by dividing the number of instances by the total number of words generated and multiplying this value by 100, with higher scores reflecting greater disorganization. We used this global score rather than individual CDI subscales because: (1) this approach is consistent with prior studies using this paradigm, facilitating comparison across studies; and (2) individual CDI categories have low base rates in two-minute samples, making subscale analyses unreliable. Transcripts were scored by multiple trained research assistants following established protocols (Minor et al., 2016). Weekly meetings were held to resolve scoring discrepancies, finalize scores, and maintain rating accuracy across raters. Interrater reliability was calculated on 20 randomly selected narratives prior to consensus meetings (ICC = 0.68). Final scores used in all analyses reflect post-consensus ratings.

#### 2.2.3 Speech production

In addition to disorganized speech, we also measured speech production, defined as the total number of words produced during each two-minute sample. Speech production was included because reduced verbal output has been associated with negative symptoms in schizophrenia, and prior studies using this paradigm have examined whether condition effects differ for disorganization versus speech quantity (Marggraf et al., 2019; Minor et al., 2016).

#### 2.2.4 Social and role functioning

The Global Functioning Scale: Social (GFS) and Role (GFR) assessed participants' real-world social and occupational functioning (Cornblatt et al., 2007). On the GFS, participants answered a series of open-ended questions about the quality of their social interactions and relationships. On the GFR, participants were asked about their engagement in productive activities, including employment, education, and household responsibilities. The assessments were administered during participants' laboratory visit by a trained clinician who was blinded to the speech assessment results. Following each interview, responses were scored using the respective ten-point scales, with each scale (GFS and GFR) producing its own separate score. A score of one indicated “severe impairment” and ten indicates “no impairment” on each scale.

#### 2.2.5 Neurocognition

Neurocognitive functioning was assessed using the Brief Assessment of Cognition in Schizophrenia (BACS; Keefe et al., 2004; Nuechterlein et al., 2008). The BACS is a widely used and well-validated measure, specifically designed for individuals with schizophrenia, with established psychometric properties (Keefe et al., 2004). The BACS produces an overall neurocognitive functioning estimate and estimates across six domains (verbal memory, working memory, motor speed, verbal fluency, processing speed, executive functioning) using age-corrected z-scores. Trained research assistants administered the BACS following standardized protocols. In our analyses, we report correlations with both the composite BACS score and the verbal memory subdomain specifically.

### 2.3 Analyses

Analyses were conducted using IBM SPSS Statistics [version 30]. Analyses proceeded in four parts. First, demographic differences between groups were assessed using *t*-tests or Chi-Square tests (*χ*^2^) as appropriate. Second, we used two analytically distinct, but complementary, approaches to measure reactivity: a) residualized change scores and b) 2 × 2 repeated-measures ANOVAs. Standardized residual change scores, following Minor et al. (2016) and Marggraf et al. (2019), were calculated by regressing speech from experimental conditions onto speech from the neutral condition. Repeated-measures ANOVAs tested group differences in mean condition effects with group (schizophrenia, control) and condition (baseline, affective/cognitive) serving as independent variables. These approaches test related, but non-identical, estimands: residualized change captures individual-level reactivity conditional on the neutral speech condition, whereas the repeated-measures ANOVA tests group differences in mean condition effects. Third, within-group Pearson correlations were computed in the schizophrenia group to assess relationships between disorganized speech and social, role, and neurocognitive functioning. These analyses were exploratory. Given the number of correlations examined (32), we applied the Benjamin-Hochberg False Discovery Rate (FDR) correction, which set the adjusted significance threshold at *p* ≤ .00156. We also report effect sizes to provide a more complete picture of findings. Finally, effect sizes were calculated using Cohen's *d* for group comparisons and partial eta-squared (ηp^2^) for ANOVA effects.

## 3 Results

### 3.1 Demographics

Independent t-tests and chi-square tests revealed that control and schizophrenia participants did not significantly differ in age, ethnicity, race, gender, or father's education level (see Table 1). However, groups differed significantly in marital status (*p* = .002), education level (*p* < .001), and mother's education level (*p* < .001). Control participants were more likely to have been married and to have completed at least a high school education. Additionally, their mothers were more likely to have completed an undergraduate or graduate degree.

**Table 1:** Participant demographics.

	SZ (n = 35)		HC (n = 37)		t/*χ*^2^
	M/N	SD/%	M/N	SD/%	
Age	42.91	11.84	42.66	11.44	−0.09
Gender					
Female	21	60.00%	23	65.71%	0.25
Male	14	40.00%	12	34.29%	
Race					
Black/AA	23	65.71%	23	65.71%	
NA/AN	1	2.86%	0	0%	
Western Asian	1	2.86%	0	0%	
White	9	25.71%	11	31.43%	
Multiracial	1	2.86%	1	2.86%	
Ethnicity					
Hispanic	0	0%	1	2.86%	1.01
Education level: self					
No HS diploma/GED	9	25.71%	0	0%	35.35***
HS diploma/GED	8	22.86%	1	2.86%	
Some college	14	40.00%	11	31.43%	
Bachelor's degree	2	5.71%	13	37.14%	
Master's degree	2	5.71%	8	22.86%	
Doctorate	0	0%	2	5.71%	
Education level: mother					
No HS diploma/GED	10	30.30%	0	0%	18.92*
HS diploma/GED	7	21.21%	11	31.43%	
Some college	5	15.15%	3	8.57%	
Bachelor's degree	8	24.24%	12	34.29%	
Master's degree	3	9.09%	8	22.86%	
Doctorate	0	0%	1	2.86%	
Education level: father					
No HS diploma/GED	6	24.00%	2	6.45%	10.47
HS diploma/GED	7	28.00%	11	35.48%	
Some college	7	28.00%	3	9.68%	
Bachelor's degree	4	16.00%	10	32.26%	
Master's degree	1	4.00%	3	9.68%	
Doctorate	0	0%	2	6.45%	
Marital status					
Never married	25	71.43%	10	28.57%	16.63**
Married	4	11.43%	16	45.71%	
Divorced or separated	5	14.29%	8	22.86%	
Widowed	1	2.86%	1	2.86%	
Symptoms					
Positive	14.91	6.83	–	–	
Negative	16.88	6.99	–	–	
Disorganized	13.48	4.37	–	–	
Hostility	6.38	3.09	–	–	
Depression	10.29	5.46	–	–	

Notes. ***p < .001, **p < .01, * *p* < .05. Symptoms measured via the Positive and Negative Syndrome Scale (Kay et al., 1987) and using the factor structure from Bell et al., 1994.

### 3.2 Reactivity

Reactivity scores were computed using the regression method described above. It was revealed that participants in the schizophrenia group showed significantly greater affective reactivity compared to healthy controls, *t*(1,70) = −3.00, *p* = .004. There was no significant difference between groups in cognitive reactivity (see Table 2).

**Table 2:** Group comparisons of disorganized speech and speech production.

	SZ (n = 35)		HC (n = 37)		*d*
	M	SD	M	SD	
Affective reactivity⁎⁎⁎	0.35	1.00	−0.31	0.89	−0.71
Cognitive reactivity	0.11	1.21	−0.10	0.75	−0.21
Neutral condition					
Disorganized speech⁎⁎⁎	2.88	1.86	1.48	1.25	−0.89
Speech production	258.74	73.06	290.30	65.63	0.46
Affective condition					
Disorganized speech⁎⁎⁎	3.57	1.83	2.02	1.65	−0.89
Speech production⁎	267.29	64.41	295.49	51.21	0.49
Cognitive condition					
Disorganized speech⁎	3.59	2.46	2.44	1.38	−0.58
Speech production⁎⁎	194.77	68.41	235.35	58.58	0.64

⁎ *p* < .05.

⁎⁎ *p* < .01.

⁎⁎⁎ *p* < .001.

### 3.3 Disorganized speech across neutral and affective conditions

**Fig. 2:**
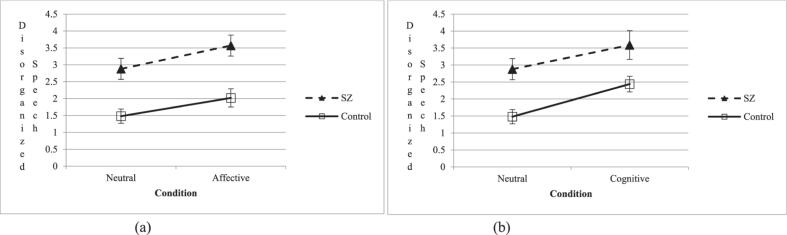
Divergent speech patterns between schizophrenia (SZ) and control groups across neutral, affective (a), and cognitive (b) conditions.

Main effects of group and condition were observed for both disorganized speech and speech production (Fig. 2). Participants in the schizophrenia group had a greater rate of disorganized speech, *F*(1,70) = 27.23, *p* < .001, *η*^*2*^*p* = .28, and produced fewer words than control participants, *F*(1,70) = 4.49, *p* = .038, *η*^*2*^*p* = .06. There was also a main effect of condition; speech disorganization was greater in the affective condition compared to the neutral condition, *F*(1,70) = 5.22, *p* = .03, *η*^*2*^*p* = .07. However, there was no main effect of condition for speech production, *F*(1,70) = 1.65, *p* = .20. Results of the repeated measures ANOVA did not support our hypothesis that there would be a significant group by condition interaction for either disorganized speech, *F*(1, 70) = 0.09, *p* = .77, or for speech production *F*(1,70) = 0.10, *p* = .75.

### 3.4 Disorganized speech across neutral and cognitive conditions

Main effects of both group and condition were again observed for both disorganized speech and speech production. Compared to control participants, schizophrenia participants exhibited greater rates of disorganized speech, *F*(1,70) = 12.74, *p* < .001, *η*^*2*^*p* = .15, and produced fewer words *F*(1,70) = 88.58, *p* < .001, *η*^*2*^*p* = .56. A main effect of condition emerged across groups for both disorganized speech, *F*(1,70) = 13.22, *p* < .001, *η*^*2*^*p* = .16, and speech production, *F*(1,70) = 6.31, *p* = .014, *η*^*2*^*p* = .08. The hypothesized group by condition interactions for disorganized speech, *F*(1,70) = 0.272, *p* = .60, and speech production, *F*(1,70) = 0.510, *p* = .48, were not supported.

### 3.5 Associations with functioning

Within the schizophrenia group, exploratory Pearson's *r* correlations showed moderate inverse associations between neurocognitive functioning and affective reactivity, cognitive reactivity, and disorganized speech in the affective condition (Table 3). These associations appear to be driven primarily by the verbal memory subdomain, which showed moderate associations with affective reactivity and disorganized speech in the affective condition. Negligible to moderate associations were observed between reactivity scores with social or role functioning (*r* = −0.07 to −0.34). After applying the Benjamin-Hochberg FDR correction, no correlation reached the adjusted significance threshold (*p* ≤ .00156). Given the consistency and magnitude of effects involving affective processes and neurocognition, these findings warrant replication in larger samples.

**Table 3:** Correlations within SZ group.

	Social Fx *r* (n = 34)	Role Fx *r* (n = 33)	BACS *r* (n = 31)	Verbal memory *r* (n = 34)
Affective reactivity	−0.25	−0.21	−0.41	−0.36
Cognitive reactivity	−0.07	−0.07	−0.36	−0.15
Neutral condition				
Disorganized speech	0.06	−0.34	−0.03	0.08
Speech production	0.34	0.28	0.24	0.08
Affective condition				
Disorganized speech	−0.24	−0.29	−0.41	−0.39
Speech production	0.06	0.04	0.13	0.16
Cognitive condition				
Disorganized speech	−0.04	−0.22	−0.34	−0.18
Speech production	0.11	0.11	0.18	0.29

Note. Correlations reached significance after FDR correction (adjusted threshold *p* < .00156). Effect sizes: small, *r* = 0.10–0.29; medium, *r* = 0.30–0.49; large, *r* ≥ 0.50.

Fx = functioning.

## 4 Discussion

This study assessed disorganized speech, affective reactivity, and cognitive reactivity in schizophrenia, and their association with functional deficits. Three key findings emerged. First, individuals with schizophrenia demonstrated significantly greater disorganized speech than healthy controls across all conditions, with disorganization increasing under affective and cognitive conditions relative to neutral across both groups. These results replicate and extend prior research, supporting the validity of the speech task and the context-sensitivity of disorganized speech. Second, regression analyses, but not repeated measures analyses, showed evidence of affective reactivity. No evidence of cognitive reactivity was observed using either method. Third, both affective and cognitive reactivity showed moderate inverse relationships with neurocognitive functioning in the schizophrenia group, but showed smaller associations with social or role functioning. This contrasts with prior work using this paradigm, which found that affective reactivity was inversely associated with social functioning in early psychosis (Minor et al., 2016) and schizotypy (Marggraf et al., 2019) samples.

The robust group differences in disorganized speech, with large effects in two of three conditions, confirm the robustness of our paradigm and align with extensive research in schizophrenia (Abel et al., 2025; Docherty, 2005; Rubino et al., 2011). The schizophrenia group also produced significantly fewer words than controls in two of three conditions, suggesting that speech disruption involves both disorganization and reduced verbal output. Moreover, both groups showed significant condition effects, with affective and cognitive manipulations increasing disorganized speech relative to the neutral condition. This demonstrates the dynamic, context-dependent nature of speech organization. These findings are consistent with prior studies using similar paradigms across the psychosis spectrum (Minor et al., 2011; Minor et al., 2016; Marggraf et al., 2019), suggesting that disorganization operates dimensionally, with schizophrenia representing heightened baseline propensity for disorganization that becomes amplified by emotional or cognitive stressors.

A central finding of this study is that affective reactivity emerged only when using a regression-based approach that controls for neutral condition disorganization. While past studies showed that negative emotions intensify disorganized speech across the psychosis spectrum (de Sousa et al., 2016; Docherty and Hebert, 1997; Minor et al., 2016; Rubino et al., 2011), affective reactivity was not detected when probing interactions using the repeated measures ANOVA. This discrepancy is methodologically important. Prior studies in schizotypy and first-episode psychosis yielded consistent results across both analytical approaches (Minor et al., 2016; Marggraf et al., 2019), but that pattern did not replicate here.

The divergent findings highlight important methodological considerations. The repeated measures ANOVA examines whether individuals with schizophrenia show disproportionately greater increases in disorganization under affective stress relative to controls. In contrast, the regression approach assesses whether emotional stress predicts increases in disorganization after accounting for neutral condition levels. This method can detect reactivity even when both groups show similar-sized increases, because it focuses on the overall effect of emotion while controlling for individual differences in starting points. That affective reactivity emerged only in the latter analysis suggests that emotional stress meaningfully impacts speech in schizophrenia, but not in a way that produces differential change across groups. These findings illustrate how analytic approaches can shape conclusions and show the value of using complementary methods to capture the multifaceted nature of speech reactivity.

In contrast to affective reactivity, we found no evidence of cognitive reactivity using either analytical approach. This aligns with several past studies that found no cognitive reactivity (Le et al., 2017; Minor et al., 2016), despite the well-established cognitive deficits in schizophrenia (Bowie and Harvey, 2006). Interestingly, both affective and cognitive reactivity were inversely associated with neurocognitive functioning within the schizophrenia group. This leads to the question: why doesn't adding a concurrent cognitive load task lead to greater disorganized speech when people with schizophrenia already show significant cognitive vulnerability? One possibility is that the dual-task paradigm used in this study, while cognitively demanding, may not have sufficiently taxed the specific cognitive systems underlying speech organization. Alternatively, individuals with schizophrenia may already be operating near cognitive capacity, such that additional task demands do not produce detectable increases in disorganization. These findings suggest the need to explore different types of cognitive tasks or more sensitive measures of cognitive reactivity in future studies.

Notably, cognition showed moderate negative correlations with cognitive reactivity in schizophrenia, supporting past findings linking cognitive dysfunction and stress sensitivity in this population (Morrens et al., 2007; Myin-Germeys et al., 2002). Cognitive functioning also showed moderate inverse correlations with affective reactivity and disorganized speech in the affective condition. This mirrors findings of attentional deficits being related to referential errors in patients in a negatively valenced affective speech condition (Burbridge and Barch, 2002). The consistency of these associations across multiple related indices (affective reactivity and affective condition disorganization both showing similar correlations with BACS and verbal memory) suggests a meaningful relationship between affective speech processes and neurocognition, though replication is needed.

Surprisingly, negligible to moderate relationships emerged between speech indices and social or role functioning. Our laboratory previously found moderate to large inverse associations between social functioning and affective reactivity and positive disorganized speech in the affective condition in both early psychosis and schizotypy samples (Minor et al., 2016; Marggraf et al., 2019). One possibility for the unexpected results here is that the larger effects seen in our previous studies are specific to earlier stages of illness and are less pronounced at later stages. This interpretation may align with recent work from Bambini et al., 2022a, Bambini et al., 2022b who observed that different types of language profiles showed differential profiles with quality of life and psychotic symptoms. This could also hold implications for identifying which clients with disorganized speech might benefit most from interventions (Bambini et al., 2022a, Bambini et al., 2022b; Minor et al., 2022). It is important to note that, in the current study, none of our findings reached the level of statistical significance once FDR correction was applied. While some observed effects represent medium associations by conventional standards (Cohen, 1990), the failure to survive correction (possibly due to modest sample size) means correlational findings should be treated as preliminary and require replication in larger samples.

This study's strengths include its experimental design, which allowed for investigation of both cognitive and affective reactivity, and its use of a behaviorally-based instrument for rating disorganized speech. However, several important limitations warrant consideration. First, the speech paradigm lacked checks to confirm that the affective manipulation was successful—though previous studies have demonstrated that similar paradigms have been successful in increasing negative affect (Cohen et al., 2010). A second limitation is the relatively brief assessments of social and role functioning. More comprehensive assessments would provide richer understanding of possible links between these variables and reactivity. A third limitation is the sample size. A larger sample would have permitted detection of smaller effects, which may be especially relevant to the correlational results.

It is also important to consider how our findings relate to naturalistic speech contexts. Our paradigm uses semi-structured speech elicitation with standardized prompts, which offers experimental control but may differ from spontaneous conversational speech. Recent advances in passive audio sensing have enabled examination of speech patterns during natural daily conversations in schizophrenia populations (Abel et al., 2025; Minor et al., 2022). Future research could examine whether the affective reactivity patterns observed in our controlled paradigm generalize to naturalistic contexts where emotional content emerges spontaneously. The growing emphasis on digital phenotyping and automated linguistic analysis (Bedi et al., 2015; Chan et al., 2023; Corcoran et al., 2018; Nikzad et al., 2025; Tang et al., 2025) provides promising tools for such investigations. It is also worth noting that disorganization is increasingly conceptualized as a pragmatic phenomenon, reflecting broader disruptions in the communicative use of language (Agostini et al., 2021; Meister et al., 2025). The combination of advancing technology and naturalistic speech sampling offers promising opportunities to examine how pragmatic aspects of disorganization unfold in real-world contexts.

### 4.1 Conclusion

This study provides new evidence that disorganized speech in schizophrenia is contextually reactive, with both affective and cognitive reactivity emerging as key correlates of neurocognitive functioning. While both the schizophrenia and control groups showed increased speech disruption under affective and cognitive load conditions, only the schizophrenia group exhibited heightened affective reactivity using residualized change scores. Understanding reactivity is critical—not only as a potential endophenotype for psychosis risk (Gottesman and Gould, 2003; Bearden et al., 2011), but also as a means of guiding personalized intervention strategies. These findings build on prior work by extending speech reactivity paradigms to schizophrenia and suggest that speech reactivity could be a clinically relevant marker of vulnerability to symptom exacerbation, as well as a potential target for personalized intervention. For instance, individuals could be matched to emotion regulation or cognitive training interventions (Penn et al., 2007; Wykes et al., 2011) based on their reactivity profiles. Future research should investigate the developmental course and real-world consequences of speech reactivity, including whether laboratory-based reactivity patterns generalize to naturalistic contexts, to refine mechanistic models and inform intervention efforts.

## CRediT authorship contribution statement

**Kyle S. Minor:** Writing – review & editing, Writing – original draft, Supervision, Project administration, Methodology, Investigation, Funding acquisition, Formal analysis, Data curation, Conceptualization. **Madisen T. Russell:** Writing – review & editing, Writing – original draft, Project administration, Methodology, Formal analysis. **Evan J. Myers:** Writing – review & editing, Writing – original draft, Project administration, Methodology, Formal analysis. **Audrey T. Satchivi:** Writing – review & editing, Writing – original draft, Methodology. **Maya E. Brown-Hughston:** Writing – review & editing, Writing – original draft, Methodology, Formal analysis. **Erica L. Whiting:** Writing – review & editing, Writing – original draft, Methodology, Formal analysis. **Deborah Daluga:** Writing – review & editing, Methodology. **Rachel C. Marks:** Writing – review & editing, Methodology. **Michaela M. Di Palmo:** Writing – review & editing, Methodology. **Basma O. Aly:** Writing – review & editing, Methodology.

## Declaration of Generative AI and AI-assisted technologies in the writing process

During the preparation of this work the author(s) used ChatGPT5 and Claude Sonnet 4 to improve the readability and edit mistakes in the manuscript. After using these tools, the author(s) reviewed and edited the content as needed and take full responsibility for the content of the published article.

## Funding

This work was supported by the 10.13039/100000002National Institutes of Mental Health [R03MH116288].

## Declaration of competing interest

We have nothing to declare.
